# Palliative Care in Portugal—From Intention to Reality, What Is Yet to Be Accomplished

**DOI:** 10.3390/nursrep13040124

**Published:** 2023-10-23

**Authors:** Paulo Marques, Francisca Rêgo, Rui Nunes

**Affiliations:** 1 CINTESIS (NursID)—RISE, Nursing School of Porto (ESEP), Rua Dr. Antº Bernardino de Almeida, nº830, 4200-072 Porto, Portugal; 2 Department of Community Medicine, Health Information and Decision, Porto Faculty of Medicine (FMUP), 4200-319 Porto, Portugal; mfrego@med.up.pt (F.R.); ruinunes@med.up.pt (R.N.)

**Keywords:** palliative care, health policy, health knowledge, education, organizational efficiency

## Abstract

**Highlights:**

**What are the main findings?**

- The flexibility of the palliative care implementation model is as or more important than its principles.
- Public education is a fundamental requirement for the dissemination of palliative care.

**What is the implication of main findings?**

- The participation of health actors is essential for higher-level decisions to be effective.
- It is crucial to invest in basic knowledge on PC in undergraduate training of health professionals and in the establishment of a medical palliative care specialty.

**Abstract:**

Objectives: This article focuses on exploring the evolution of palliative care in Portugal. Ten years after the approval of its Basic Law, the aim was to investigate the quality of the path followed and the guidelines that could promote its development. Thus, this study sought to identify (a) the goals of the current members of parliament concerning palliative care, (b) the major priorities that should guide the development of palliative care in the coming decade, (c) the facilitating or hindering factors to accelerating the process, and (d) to propose consensually agreed measures for the integral development of palliative care within the health system. Methods: The qualitative data analysis was performed through the reading of the literature and interviews conducted via Zoom with several intentionally chosen participants. The data extracted from the previous studies were analyzed in a focus group. The NVivo^®^ 10 was used for the data processing and categorization. Results: Three key themes emerged concerning the current status of palliative care: the policymakers, the health professionals, and the society. This first line of structuring is explained by a second set of categories, namely, (a) the knowledge about palliative care; and (b) palliative care organization from the policymakers’ perspective. In the health professionals’ domain: (a) knowledge about palliative care, (b) clinical training, and (c) medical specialty. Finally, in society: (a) knowledge about palliative care. Significance of the results: Advancing general education, increasing the qualitative training for different health professionals, reformulating the laws supporting them, promoting the flexibility of the implementation methodologies, and establishing a medical specialty are crucial to achieving the proposed goal. This study was not registered.

## 1. Introduction

According to the World Health Organization (WHO), each year, about 56.8 million people need palliative care (PC) worldwide. Access to PC is limited to about 12%, with needs continuing to increase due to aging and escalating chronic diseases, estimated to double by 2060 [1]. The delivery of PC is an ethical responsibility of health systems, benefiting individuals directly and systems indirectly. The access to PC is globally considered an important factor in the control of cancer by 2025 [2].

In Portugal, we are already witnessing significant advancements, especially after the publication of the Basic Law in palliative care—Law n. º 52/2012 [3], dated September 5. However, since a decade has elapsed since this publication, it is vital to assess what has been achieved to decide where to go next.

Thus, two research questions were formulated: (a) what is yet to be accomplished? and (b) what is needed to ensure this accomplishment in due time? In leading the process of building a whole organization that aims to respond to the PC needs, the National Palliative Care Commission (NPCC) under the Directorate-General of Health (DGS) has been preparing biennial plans since 2017–2018 [4,5,6], producing reports [7,8] which reflect its perspective on progress and what is needed to accomplish this progression. Moreover, an independent analysis of PC development in Portugal was considered important.

Portugal follows a trend of fruitful legislation on different topics, placing the difficulties at lower levels of operationalization encompassing the will, structure, or existence of the necessary material and human resources. The provision of PC is not limited to specialized services but to other more general services integrated into the health system, also seeking the relief of suffering and symptom control [9]. There is a need for trained health professionals to provide specialized care in this area worldwide [10]. Chronic diseases are closely related to PC needs and are highly prevalent in most developed or developing countries. In addition, the onset of new chronic diseases caused by COVID-19 and the increasing aging population adds a substantial burden to this reality. In Portugal, the last census (2021) points to 23.4% of the elderly population and its progressive increase [11]. PC is an approach that improves the quality of life of patients (adults and children) and their families who are facing problems associated with life-threatening illness. It prevents and relieves suffering through the early identification, correct assessment, and treatment of pain and other problems, whether physical, psychosocial, or spiritual [12]. This will have repercussions in reducing the use of health services and consequent expenses, with a relationship between the different vectors. However, the problem is not always about resources but the management of these resources. This is highly debated in Portugal, often referring to the differences between the public and private sectors.

The Strategic Development Plan for Palliative Care 2021–2022 [6] pointed to various obstacles to implementing the plan. It also recognized difficulties and highlighted essential aspects already put into practice, anticipating no significant evolution, especially due to the pandemic outbreak. This plan stresses that many Portuguese people still do not have access to PC due to different factors and the geographical distribution of the population, with inequalities between those who live near the coastline or in more rural areas [13], common to many other resource distributions. Internationally, it is considered that the challenge to the growth of PC responses is related to the limitations of financial and material resources, knowledge, a limited number of training courses, and a lack of public awareness.

Thus, this research sought to focus on (a) the awareness of the population versus health professionals—crucial for awareness of the problem and referral, (b) the training at different levels and lengths—for the promotion of qualified professionals, and (c) the dissemination of support teams and implementation of PC—referring to human resources and infrastructures.

In this perspective, we seek to respond to the following objectives:

- To identify the priorities that should be used as guidelines for development in the next decade.
- To identify the facilitating and hindering factors for advancing the process.

## 2. Methods

A qualitative methodological approach was considered to meet the objectives outlined in this research. This method was preferred over quantitative dynamics because exploring such a complex topic as PC would mostly benefit from the participants’ discourse. Hence, this analysis was framed by three axes, namely: (1) the policies based on the programs of the political parties with parliamentary seats; (2) the priorities guiding PC for the next decade (2022–2032); and (3) the identification of factors hindering the PC development process in Portugal.

Qualitative research focuses on the reality of people, events, and places. It does not transform information into numerical symbols but attempts to analyze it in all its richness. Thus, words are crucial for recording the information and disseminating the results. Qualitative research is descriptive, enabling it to include all aspects of the evaluation. In this type of research, the researcher does not assume to have all the knowledge about the important topics of the study. Thus, the uncovering of relevant material is achieved while gathering the information.

In view of this assumption, a qualitative observational study was conducted, centered on documental analysis. The first phase addressed the revision of the diplomas and official reports framing the legislative structure and state of the art, and the political agenda of the elected politicians and others on the guidelines concerning palliative care (of public access); the second phase considered the vision of the different intervening parties in the process and the opinion of prominent persons in palliative care, namely referring to health professionals; and, finally, the third phase included a focus group to engage in discussions based on the synthesis of the data previously gathered, intending to increase the relevance of the information already obtained in the previous study. The inclusion criteria included the following: (a) a member of a political party with a parliamentary seat or not (exceptionally), (b) a member of civil society organizations focused on PC, (c) a member of an official organization of the Ministry of Health in the area, (d) physicians and nurses, who could contribute to the topic under study, covering the largest geographic area. The sample of health and teaching professionals was recruited among experts working in palliative care. The focus group included six experts.

Interviews were conducted via a video call from Porto using Zoom© software. The study design was organized into three sequential studies:

Documentary analysis (international guidelines, political guidelines—legislation—and party ideologies, guidelines of the national associations linked to PC, NPCC reports, among other resources).

Semi-structured (recorded) interviews with representatives (experts) of political parties and subject matter experts from clinical practice and health education—discourse content analysis.

A focus group with a restricted number of experts to analyze the preliminary results obtained.

Permission was sought to record the interviews, and the data were stored and processed using NVivo© 10 software for categorization following the General Data Protection Regulation [14]. The data obtained were encrypted using keys specially created for this purpose by the principal investigator (PI). The PI had full and exclusive access to the data to be destroyed after the end of the study.

The interviews were conducted according to a script with questions addressing the characterization of palliative care politics, a reflection on equity in PC access, the difficulties in establishing a care network adequate to the needs and the reasons supporting them, and the proposals for improving the current framework.

The readings of the corpus from which the expressions were extracted, constituting semantic units, were followed by a categorization with an in-depth comprehension of the expressions through a central themes analysis, using a predominantly descriptive narrative.

The rigor and validity of the research with these characteristics are derived from the criteria inherent to the careful process of the treatment, review, and validation. Therefore, the focus group and the documentary analysis, which translates multiplicity and the use of triangulation of methods, adding security and robustness, were selected as the best methodologies for this research.

This research followed all the required ethical procedures and was approved by the Ethics Committee of the Faculty of Medicine of the University of Porto—Opinion 54/CEFMUP/2022 of 28 July 2022.

## 3. Results

A total of 16 interviews were conducted from September to November 2022, lasting about 42 min. Of the interviews, 11 were conducted with physicians, 3 with nurses (two were higher education teachers), and 2 interviews were conducted with political representatives. The respondents included “anonymous” persons and others with relevant experience in PC and currently and previously integrating organizations linked to palliative care, professional associations, and NPCC. The participants working in clinical practice belong to structures all over the mainland, coastal, and inland, which consisted of the three types of units established by law, namely, PC inpatient units, PC in-hospital support teams, and PC community support teams.

After processing the initial data, the focus group was carried out with five intentionally chosen professionals, persons with merit in PC, four nurses (two from teaching), and one physician, for the analysis and discussion of the global results already produced. This method lasted about two hours.

Excerpts from the interviews will be coded by the letter “E” followed by the categorization number, similar to the method to be used for the focus group.

**Figure 1:**
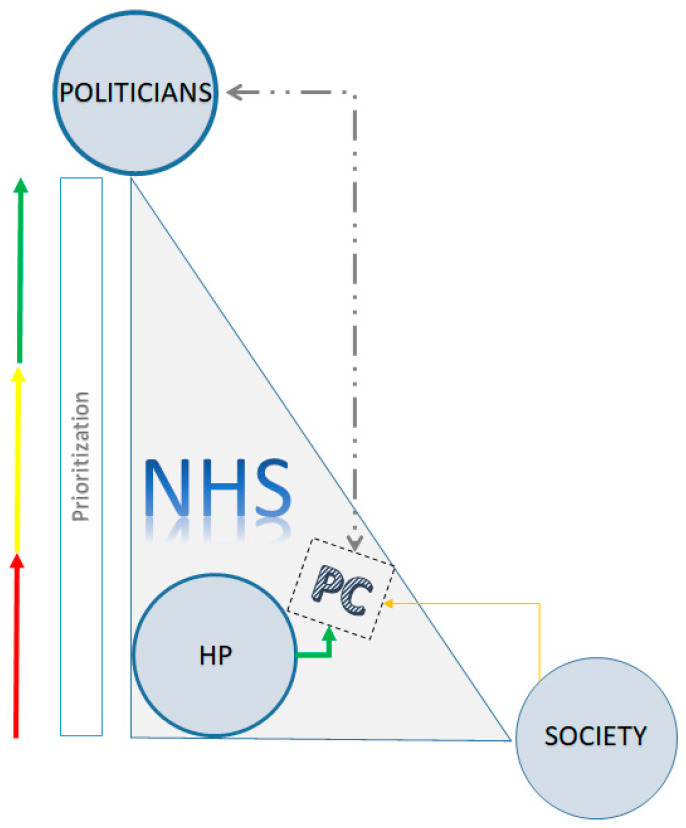
The triangle of forces where the positioning of palliative care is displayed.

From the data analyzed in the first instance, three major themes emerged that structured and gave meaning to the PC state of the art in Portugal, as shown in this research’s results: (a) the politicians (P): “they don’t understand (the politicians) what we do, what PC is” (E1), which somehow by action or omission tend to facilitate or hinder developing a health care dimension; (b) the health professionals (HP): “the dynamics are from bottom to top. People (professionals) want and make it happen” (E3), embodying care and stimulating its development; and (c) the society (S): “there is a total lack of knowledge (from the society) of what PC is” (E6) which uses the resources and can influence decision-making but has little knowledge about palliative care. Figure 1 aims to schematically represent this classification. These three pillars are at the apexes of a scalene triangle since each has distinct “weighting” in the constructing and solidifying of PC development within the National Health Service. In this relationship of forces, HP have been the driving force to a more consistent and continuous prioritization and in the leveraging of PC as an important integrated part of the health system. On the other hand, the political agenda has been discontinuous, varying its pace according to the health professionals’ purposes, who in some cases occupy intermediate and upper management roles. Society has neither been particularly demanding concerning this area nor active in claiming more and better offers: “only a society that demands differently will get it” (E3).

Even with different levels of intensity, there is one dimension transversal to the three pillars mentioned, that of “knowledge about palliative care”: “(knowledge about PC) is the major problem for all, not acknowledging what is at stake” (FG2).

According to participants, a significant part of the challenges posed to the development of palliative care in Portugal is related to a lack of understanding of the concept and its attributes: “today, I was approached by a professional saying that one patient needed us, but she was too young…” (FG2). This can also result in refusal to receive this type of care: “there is only a reason for the patient not wanting, and this is for lack of knowledge” (FG2). On the other hand, politicians tend to associate the concept with the number of beds: “there is a misunderstanding in associating PC patients to beds” (FG3). In the programs of the political parties stricto sensu, there is an absence of substantial and distinctive elements, highlighting the association with “answers in terms of beds”. This is related to the “began with inpatient units leading people to associate PC with a specific place, not a type of care” (E14). The lack of knowledge about this topic and policy decision-making based on “political agenda” (E6) does not place this issue on top of the priorities.

In PC organization, the legislation, structure, and functioning of the National Health Service also do not contribute to the adequacy of responses to PC needs. The biomedical model is still the most preferred: “everything is centered on hospital and emergency services” (E14), and difficulties have emerged because the Basic Law in Palliative Care is dissociated from the Basic Law in Health: “the DGS statements reach more professionals than the Commission, giving an idea that PC does not involve everyone work” (E1).

On the other hand, the biannual strategic plans and the commission responsible for their development present several limitations hindering the results, such as timing: “strategic plans are difficult to achieve, they should last five years” (E12), the focus, because they are: “little ambitious, having more teams does not guarantee anything” (E3), the rigidity of the model: “the model we have is the same for the entire country without considering local aspects” (E5); the decision power: “the CNCP has no authority, and it’s not clear its enforcing role, so it cannot impose a global strategy” (E13), and the lack of knowledge of the problems: “the organization lacks field knowledge” (E1).

Moreover, the teams need to follow a set of guidelines concerning the number of professionals and houses, that often are not fulfilled: “there are assigned teams, but professionals have neither a schedule, nor training and resources” (E14); on the other hand, they lack guidance: “they work according to what they perceive is the best and there is an assumption about quality” (E13), missing indicators “to assess its functioning and assess what is needed to improve” (E13); there are populations with access to resources and others that lack these resources, even with geographical proximity: “the teams are limited in human resources and geographical areas, following an administrative logic” (EP1), and with the duplication of resources: “some hospitals assign their home team and if there is a community team, this might duplicate resources” (E13); and there is neither a timely response nor do these resources meet the amplitude of the needs: “if there’s a waiting list, prioritizations are needed because most of our patients need to be accompanied in their last days of life” (E8); and even when there are available resources they are not operational all the time: “families report to us about the importance of support over the weekends” (E8), stressing the need to “discuss the assistance model” (FG4), ultimately transforming it into “flexible models and plans” (FG4). Concerning the health professionals, training is viewed as a key element for the proposed development, including “a major investment in pre-training, with a specific and clinical discipline. Medicine Faculties are already adopting this strategy” (E10) because “most professionals work with palliative patients but don’t have specific training” (E3); thus, it is vital that “all professionals have minimum training” (E9) with a strong practical component: “we need to invest in training but considering competencies development” (FG5). Within the medical scope, the lack of a specialty poses an obstacle to the attractiveness of PC: “professionals wishing to dedicate themselves to this area need to work under certain conditions that do not interfere with their performance” (E14), thus, the existence of a specialty in PC will highly contribute to the development of palliative care: “acknowledging PC as a medical specialty would substantially contribute to increase responses” (E11).

## 4. Discussion

In Portugal, PC experienced significant development ten years ago, so it was important to assess its results. All actors, global or regional, should invest in evidence, which will facilitate policy adaptations and ensure effective progress [15] because adequate and informed changes can only be proposed with robust data. Despite several difficulties, Portugal has a track record of merit in health and disease care. This research coincided with some relevant changes at this level, namely the publication of the new NHS Statute [16], demonstrating that it is in a transition phase, seeking the adoption of measures to improve the system.

Politicians, health professionals, and people in general need to be educated about the concept of this care and its benefits; palliative care is a human right [17]. Education should focus on non-health professionals who have greater gaps in knowledge about PC [10] and on the general population who have knowledge deficits [18]. Moreover, health courses and continuing education, which reveal greater gaps [19], should be optimized to provide professionals with skills to identify needs, refer patients to specialized services, and deliver quality targeted care [20,21]. The barrier to knowledge about palliative care is important, and there is a need to implement community education and awareness actions in parallel with the establishment of services [1]. PC is still associated with negative elements [22], with PC being a different way of approaching life [1,23]. For most people, PC is associated with end-of-life and death, thus creating a barrier to the development of PC. Moreover, psychologically, most people fear and avoid any issue related to death, and there is often a belief that just acknowledging the possibility of someone dying is harmful [1]. Internationally, there is a lack of interest in PC despite it being considered an essential component of universal health provision. This might be explained by the less compatible results with those that drive policy and investment (such as prolonging life and productivity), the focus on cure (biomedical conception) and neglect of care that promotes quality of life near the end of life [15]. Notably, professionals, patients, and families convey a positive image of this care [22].

Developing PC education and awareness campaigns should be considered a public priority [15]. This aspect is also detailed in the different National Strategic Plans [6].

Among health professionals, prejudice about PC persists, which is another barrier recognized by the WHO worldwide [1]. This aspect is rooted in the curative approach model and goes beyond it; however, despite the training, there is not always an incorporation of the paradigm that governs this type of care.

Furthermore, medical and nursing training has gone through a substantial evolution. The number of medical and nursing schools with units in undergraduate curricula is a central and strategic indicator for PC development [24], and the global health workforce gap will be mitigated if all students who are graduating receive specific training, improving access to care [1,17].

An important contribution to pre or postgraduate training, is clinical training, which is also dependent on adequate contexts for its development and the quality of care and outcomes. Those who provide palliative care should be trained to acquire optimal skills [17,25]. Without adequately trained people and sufficient environments, progress cannot be made as quickly as desired.

As for the PC specialty, the biggest issue is in the medical sphere rather than nursing because the greatest challenge is the need to create a medical specialty in this area [24], and this is a key indicator for its development [24,26]. The Strategic Plan of 2021–2022 embodies this need by referring to it as a “special area of competence” [6].

When analyzing in depth the Basic Law in Palliative Care, the functioning of the National Commission, the Strategic Plans and the Reports, criticisms are pointed out in the model about its rigidity regarding changes that may lead to health gains under the current circumstances. Moreover, there are not sufficient professionals for fieldwork, covering the whole territory and all its needs; therefore, it will be possible to do more with new dynamics, rationalizing the resources, which differ according to the geographical areas and specific contexts.

The existence of gaps in the responses provided by the system emerges as notorious, warrants a reformulation [27], thus, the system must adapt to the needs arising from an increasingly aging population, particularly in proximity to palliative care, by keeping people in their natural environment [13]. The publication of the new NHS statute created the role of the Chief Executive Officer to overcome other problems, and this can potentially be a positive outcome for the development of PC [16]. article 9 in this document refers to the coordination of the support response from the SNS—National Health Service and RCNP—National Network of Palliative Care. The 2021–2022 NPCC [6] notes “organization and coordination” as the first general strategic line, with problems identified at that level. Although Portugal is technically positioned in the group of European countries with better PC coverage [9], the reality shows a different dimension. According to the Autumn Report of the Portuguese Observatory for Palliative Care, the medical coverage in PC responded only to 13.3% (28.6% according to the NPCC) of the recommended needs. Moreover, in nursing, it reached 10.2% (34.8% according to the NPCC), with percentages lowering in psychology and social work, noting that structures and professionals in the national territory are “far below the minimum acceptable” [28]. It is also essential to deepen the coordination of the multi-sectoral actors and entities dedicated to PC through the respective ministries of health [15].

## 5. Conclusions

In Portugal, there is extensive legislation on the subject, which was developed to combat problems affecting the development of PC. However, on the other hand, the “separation” of PC from other forms of health care constitutes a hindering factor that needs re-evaluation. The models in use have proved to be rigid and insufficiently adaptable to specific territorial characteristics. There is also evidence of inefficient coordination, which should be reconsidered. Finally, an investment in the general knowledge about palliative care, undergraduate training, and the establishment of a medical specialty have emerged as key factors to the Portuguese strategy in healthcare. Therefore, it is strongly recommended to make the proposed models more flexible, to take a greater role in coordinating the PC development process, and to focus on health literacy.

## Data Availability

The data presented in this study are available on request from the corresponding author.

## Public Involvement Statement

There was no public involvement in any aspect of this research.

## Guidelines and Standards Statement

This manuscript was drafted against the reporting guidelines and citation for Qualitative Research for Standards for reporting qualitative research: a synthesis of recommendations.
